# Variable Bayesian-Based Maximum Correntropy Criterion Cubature Kalman Filter with Application to Target Tracking

**DOI:** 10.3390/e27100997

**Published:** 2025-09-24

**Authors:** Yu Ma, Guanghua Zhang, Songtao Ye, Dou An

**Affiliations:** 1 School of Electronics and Control Engineering, Chang’an University, Xi’an 710018, China; 2 MOE Key Laboratory for Intelligent Networks and Network Security, Faculty of Electronic and Information Engineering, Xi’an Jiaotong University, Xi’an 710049, China; ghzhang2018@mail.xjtu.edu.cn (G.Z.); songtaoye@stu.xjtu.edu.cn (S.Y.); douan2017@xjtu.edu.cn (D.A.)

**Keywords:** target tracking, nonlinear processing technique, cubature Kalman filter, maximum correntropy criterion, variational Bayesian, non-Gaussian noise

## Abstract

Target tracking in typical radar applications faces critical challenges in complex environments, including nonlinear dynamics, non-Gaussian noise, and sensor outliers. Current robustness-enhanced approaches remain constrained by empirical kernel tuning and computational trade-offs, failing to achieve balanced noise suppression and real-time efficiency. To address these limitations, this paper proposes the variational Bayesian-based maximum correntropy criterion cubature Kalman filter (VBMCC-CKF), which integrates variational Bayesian inference with CKF to establish a fully adaptive robust filtering framework for nonlinear systems. The core innovation lies in constructing a joint estimation framework of state and kernel size, where the kernel size is modeled as an inverse-gamma distributed random variable. Leveraging the conjugate properties of Gaussian-inverse gamma distributions, the method synchronously optimizes the state posterior distribution and kernel size parameters via variational Bayesian inference, eliminating reliance on manual empirical adjustments inherent to conventional correntropy-based filters. Simulation confirms the robust performance of the VBMCC-CKF framework in both single and multi-target tracking under non-Gaussian noise conditions. For the single-target case, it achieves a reduction in trajectory average root mean square error (Avg-RMSE) by at least 14.33% compared to benchmark methods while maintaining real-time computational efficiency. Integrated with multi-Bernoulli filtering, the method achieves a 40% lower optimal subpattern assignment (OSPA) distance even under 10-fold covariance mutations, accompanied by superior hit rates (HRs) and minimal trajectory position RMSEs in cluttered environments. These results substantiate its precision and adaptability for dynamic tracking scenarios.

## 1. Introduction

High-precision state estimation serves as the fundamental cornerstone for ensuring system reliability of dynamic perception and autonomous systems. As intelligent devices are increasingly deployed in complex environments, sensor data are subject to multifaceted effects, including nonlinear propagation characteristics, multi-source heterogeneous noise, and dynamic target interactions [1,2]. Particularly in target tracking scenarios, radar may produce outliers or noise spikes due to environmental interference or hardware malfunctions [3], while adversarial electronic warfare tactics in military applications can introduce extreme noise through deliberate jamming signals [4]. Such scenarios often exhibit heavy-tailed noise distributions, deviating significantly from Gaussian assumptions [5]. Consequently, nonlinear filtering algorithms capable of mitigating these adverse factors constitute the core technology for target tracking, with their performance directly influencing the accuracy and reliability of tracking outcomes.

In recent years, nonlinear filtering techniques have evolved significantly through derivatives of the Kalman filter framework, particularly in addressing complex dynamic systems and high-dimensional nonlinear problems. The extended Kalman filter (EKF) [6,7] linearizes nonlinear models via first-order Taylor expansions within the Kalman framework. However, its reliance on local linearization introduces truncation errors that accumulate in strongly nonlinear scenarios, often leading to filter divergence. Apart from linearization errors, the vulnerability of EKF to environmental disturbances further limits its robustness. Recent innovations address this by integrating reliable measurement selection mechanisms with EKF frameworks [8]. In addition, to address these limitations, probabilistic approximation-based methods such as the unscented Kalman filter (UKF) [9,10,11] and cubature Kalman filter (CKF) [12,13] have emerged. The UKF transmits statistical moment information via unscented transform sampling of Sigma points, thereby enhancing adaptability for strongly nonlinear systems. Nevertheless, high-dimensional sampling can lead to issues of non-positive definite covariance matrices, resulting in numerical instability and degraded precision. In contrast, CKF employs Gaussian-weighted integration rules and approximates nonlinear distributions using the spherical–radial cubature criterion. By ensuring computational efficiency and designing symmetric sampling points, it enhances filtering robustness. In scenarios with higher state dimensions or greater nonlinear intensity, its estimation accuracy and robustness surpass those of the UKF, making it an essential theoretical tool for state estimation in complex dynamic systems. However, these nonlinear variants of the Kalman filter are predicated on the assumption of Gaussian noise. In heavy-tailed noise environments, where higher-order statistical moments dominate, such methods often fail to mitigate error accumulation, ultimately leading to estimation divergence.

State estimation under heavy-tailed noise conditions remains a critical challenge in dynamic systems, with existing approaches primarily focusing on noise statistical modeling and distribution approximation. Heavy-tailed distribution modeling explicitly constructs non-Gaussian noise profiles to enhance robustness [2,5,14], yet its non-conjugacy leads to the lack of closed-form solutions, necessitating moment-matching approximations that introduce additional errors. Multi-model approaches, such as Gaussian mixture models [15] and conditioned Markov switching models [16], describe noise mutation characteristics through weighted multimodal distributions. However, their practical utility is constrained by model complexity and sensitivity to predefined weights. Monte Carlo methods, exemplified by particle filters [17,18], theoretically achieve arbitrary distribution approximation via stochastic sampling. Despite asymptotic optimality, their computational complexity in high-dimensional scenarios renders them impractical for real-time applications. Beyond conventional heavy-tailed modeling, recent advances have developed dual-path adaptive frameworks to simultaneously address non-Gaussian noise in system dynamics and measurement models. A representative example is the Mahalanobis distance-based CKF with both adaptability and robustness for tightly coupled GNSS/INS integration [19]. Collectively, the trade-offs among dynamic noise adaptation, computational efficiency, and theoretical closed-form solutions remain significant challenges for these methods. Recent advancements in information-theoretic learning (ITL) have introduced novel paradigms for enhancing filter robustness by reconstructing optimization objectives through higher-order statistics and nonlinear similarity measures (such as correntropy) [20]. Unlike minimum mean square error (MMSE)-based methods that rely solely on second-order moments, the maximum correntropy criterion (MCC) embeds local similarity into the cost function through kernel function mapping, using second-order and above moments of the innovation to effectively suppress outliers while capturing higher-order data characteristics [21,22,23]. Beyond traditional filtering, the MCC framework has been extended to specialized domains requiring robust non-Gaussian noise suppression. A state-of-the-art application is spectral redshift navigation [24] leveraging MCC to dynamically estimate stellar spectral shifts under cosmic noise. Such advancements underscore MCC’s potential in enhancing robustness for complex nonlinear systems beyond filtering designs. Although MCC sacrifices closed-form solutions for recursive estimation [20], its dynamic adaptability to heavy-tailed non-Gaussian noise has demonstrated unique advantages in complex scenarios, driving its adoption in robust adaptive filtering designs [20,25,26,27,28,29]. Notably, a critical parameter in MCC-based filters is the kernel size for adjusting the gain matrix, which governs outlier suppression efficacy and estimation stability [20,25]. Current kernel size selection strategies predominantly rely on predefined static values (e.g., empirically fixed via offline experiments [20,26,27]) or online empirical adjustments (e.g., heuristic calculations based on innovation covariance [25,28,29]). However, static methods exhibit strong scenario dependency, limiting generalizability across diverse noise characteristics, while online strategies fail to adapt to abrupt interference intensity variations due to fixed adaptation mechanisms. Although a kernel size optimization mechanism based on sliding-window heuristics [30] has been proposed, its noise adaptability remains constrained by predefined window parameters, exhibiting inherent latency in responding to abrupt noise disturbances. Fundamentally, existing approaches lack an adaptive association mechanism between kernel size and dynamic noise statistics, hindering real-time adaptability in scenarios with time-varying noise distributions or sudden changes in interference intensity. Furthermore, current research predominantly targets single-objective scenarios, lacking extended modeling for multi-target complex environments. These limitations subject existing methodologies to dual challenges of degraded estimation precision and computational efficiency imbalance in scenarios involving abrupt noise characteristic transitions or multi-target interactive environments.

To address these challenges, variational Bayesian (VB) methods have emerged as a promising framework for handling complex posterior distributions and joint estimation problems [31,32,33,34]. By probabilistically modeling latent variables (e.g., kernel size) and leveraging conjugate prior properties, VB enables joint Bayesian inference of states and parameters. Building on this foundation, this paper innovatively proposes a dynamic joint estimation mechanism for kernel size and state by integrating variational Bayesian inference with the theory of MCC. Additionally, it incorporates cubature transformation techniques to minimize approximation errors in nonlinear systems. Consequently, the variational Bayesian-based maximum correntropy criterion cubature Kalman filter (VBMCC-CKF) is proposed, showcasing enhanced optimization capabilities. The key innovations are reflected in the following three aspects.

(1) Integration architecture of VB and maximum correntropy criterion cubature Kalman filter (MCC-CKF):

For the first time, this method combines VB inference with MCC-CKF to construct a joint estimation framework of state and kernel size, breaking the dependence of traditional filtering algorithms on Gaussian noise assumptions and fixed kernel parameters. By synchronously optimizing the posterior distribution of the state and the kernel size parameters, it achieves fully adaptive robust estimation under nonlinear and non-Gaussian noise.

(2) Adaptive kernel size dynamic adjustment mechanism based on conjugate distribution:

A probabilistic modeling paradigm is introduced by treating the kernel size as an inverse gamma-distributed random variable [35]. Leveraging the conjugate relationship between Gaussian and inverse gamma distributions, a closed-form analytical solution is derived through a variational Bayesian online alternating optimization. This mechanism significantly enhances robustness against heavy-tailed noise and outliers while maintaining linear computational complexity for parameter updates, ensuring real-time applicability.

(3) Scalability in single/multi-target tracking scenarios:

VBMCC-CKF demonstrates exceptional generalizability across tracking scenarios. In single-target benchmarks, it delivers superior robustness against complex disturbances. When extended to multi-target tracking via a multi-Bernoulli filtering framework [36,37,38], the method dynamically balances multi-dimensional noise suppression and computational efficiency. This enables precise continuous tracking in cluttered environments with time-varying target interactions, providing a modular solution for advanced perception systems.

The structure of this paper is arranged as follows: Section 2 systematically expounds the theoretical framework of CKF based on MCC, highlighting the limitations of conventional approaches and motivating this research. Section 3 details the proposed adaptive kernel optimization methodology, focusing on the variational Bayesian iterative framework and its mathematical derivations. Section 4 theoretically analyzes robustness mechanisms and evaluates computational complexity. Section 5 validates the algorithm through comparative simulations: single-target benchmark evaluations followed by multi-target simulations under dynamic clutter. Section 6 concludes with research summaries and future directions in nonlinear non-Gaussian systems.

## 2. Problem Formulation

### 2.1. Principle of CKF

Consider a nonlinear dynamic system [29]:

(1) $$\begin{array}{l} x_{k}=f(x_{k-1})+w_{k}\\ y_{k}=h(x_{k})+\nu_{k} \end{array}$$

where $x_{k}$ denotes the state vector at time k, $y_{k}$ denotes the measurement vector at time k, f(·) and h(·) are the nonlinear state transition and measurement functions, respectively. The process noise $w_{k}∼N(0,Q_{k})$ and measurement noise $v_{k}∼N(0,R_{k})$ are mutually uncorrelated white Gaussian noise with covariance matrices $Q_{k}$ and $R_{k}$, respectively. CKF employs a set of deterministic cubature points to approximate the posterior mean and error covariance of the state under nonlinear transformations with additive Gaussian noise, thereby enabling robust state estimation $x_{k}$ through effective exploitation of noise-contaminated measurement information.

The CKF algorithm initializes with the prior state estimate $x_{0}$ and covariance $P_{0}$, followed by recursive execution of two steps: prediction and update.

**Prediction Step:** At time k−1, given the posterior state estimate ${\hat{x}}_{k-1}$ and covariance $P_{k-1}$, generate 2n symmetric cubature points:

(2) $$\left\{\begin{array}{ll} \chi_{i,k-1}={\hat{x}}_{k-1}+\sqrt{n}\cdot {\left(\sqrt{P_{k-1}}\right)}_{i}, & i=1,\ldots ,n,\\ \chi_{i+n,k-1}={\hat{x}}_{k-1}-\sqrt{n}\cdot {\left(\sqrt{P_{k-1}}\right)}_{i}, & i=1,\ldots ,n \end{array}\right.$$

where $\sqrt{P_{k-1}}$ is the Cholesky decomposition of $P_{k-1}$. Propagating these points through f(·) yields the transformed cubature points $\chi_{i,k|k-1}=f(\chi_{i,k-1})$, i=1,…,2n. The predicted state mean ${\hat{x}}_{k|k-1}$ and covariance $P_{k|k-1}$ are computed as follows:

(3) $${\hat{x}}_{k|k-1}=\sum_{i=1}^{2n}\frac{1}{2n}\chi_{i,k|k-1},$$

(4) $$P_{k|k-1}=\frac{1}{2n}\sum_{i=1}^{2n}\chi_{i,k|k-1}\chi_{i,k|k-1}^{T}-{\hat{x}}_{k|k-1}{\hat{x}}_{k|k-1}^{T}+Q_{k-1}.$$

**Update Step:** Propagate the predicted cubature points through h(·) to generate the measurement cubature points $Y_{i,k|k-1}=h\left(X_{i,k|k-1}\right)$, i=1,…,2n. The predicted measurement mean, measurement covariance, and cross-covariance are calculated as follows:

(5) $${\hat{y}}_{k|k-1}=\frac{1}{2n}\sum_{i=1}^{2n}Y_{i,k|k-1},$$

(6) $$P_{yy,k}=\frac{1}{2n}\sum_{i=1}^{2n}\left(Y_{i,k|k-1}-{\hat{y}}_{k|k-1}\right){\left(Y_{i,k|k-1}-{\hat{y}}_{k|k-1}\right)}^{T}+R_{k},$$

(7) $$P_{xy,k}=\frac{1}{2n}\sum_{i=1}^{2n}\left(\chi_{i,k|k-1}-{\hat{x}}_{k|k-1}\right){\left(Y_{i,k|k-1}-{\hat{y}}_{k|k-1}\right)}^{T}.$$

Calculate the Kalman gain as follows:

(8) $$K_{k}=P_{xy,k}{\left(P_{yy,k}\right)}^{-1}.$$

Finally, the estimate and covariance of the posterior state are updated via the following:

(9) $${\hat{x}}_{k}={\hat{x}}_{k|k-1}+K_{k}\left(y_{k}-{\hat{y}}_{k|k-1}\right),$$

(10) $$P_{k}=P_{k|k-1}-P_{xy,k}\left(P_{yy,k}\right)P_{xy,k}^{T}.$$

### 2.2. Principle of MCC

For the random variables X, Y∈ℝ, the correntropy quantifies their generalized similarity and is defined as follows [29]:

(11) $$V_{\sigma_{k}}(X,Y)=E\left[G_{\sigma_{k}}(X-Y)\right]=\;\iint_{x,y}G_{\sigma_{k}}(x-y)dF_{X,Y}(x,y),$$

where $E\left[\cdot \right]$ denotes the expectation operator, and $F_{X,Y}(x,y)$ is the joint probability density function. Because the Gaussian kernel function can approach the nonlinear model infinitely, the kernel function $G_{\sigma_{k}}(\cdot )$ satisfying Mercer’s theorem is designed, with the kernel size $\sigma_{k}$ adjusted dynamically and adaptively:

(12) $$G_{\sigma_{k}}(x-y)=\frac{1}{{\left(2\pi \sigma_{k}^{2}\right)}^{1/2}}\exp\left(-\frac{||x-y|{|}^{2}}{2\sigma_{k}^{2}}\right).$$

By expanding the Gaussian kernel function in Equation (12) into a Taylor series and substituting it into (11), we have the following:

(13) $$V(X,Y)=\frac{1}{{\left(2\pi \sigma_{k}^{2}\right)}^{1/2}}\sum_{n=0}^{\infty }\frac{{\left(-1\right)}^{n}}{2^{n}\sigma_{k}^{2n}n!}E\left[{\left(X-Y\right)}^{2n}\right].$$

It is evident that correntropy constitutes a weighted sum of even-order moments of X−Y, thereby incorporating higher-order statistical information beyond the second moment. When $\sigma_{k}$ is sufficiently large, correntropy is determined by the second moment. Furthermore, due to the general unavailability of $F_{X,Y}(x,y)$ and the finite samples $\left\{x_{i},y_{i}\right\}$, i=1,2,…,N, correntropy is empirically estimated using the sample mean.

(14) $$\hat{V}(X,Y)=\frac{1}{N}\sum_{i=1}^{N}G_{\sigma_{k}}\left(x_{i}-y_{i}\right).$$

Let $x_{i}=g(\vartheta )$ denote the model output that depends on the parameter vector ϑ, and $y_{i}$ is the desired response. The residual term $e_{i}(\vartheta )=x_{i}-y_{i}$ depends on ϑ. MCC is defined as the optimization framework that identifies the optimal parameter vector ϑ from a feasible set Ω by maximizing the empirical correntropy in (14), formally expressed as follows:

(15) $$\vartheta =\underset{\vartheta \in \Omega }{\mathrm{argmax}}\frac{1}{N}\sum_{i=1}^{N}G_{\sigma_{k}}\left(e_{i}(\vartheta )\right).$$

In practical engineering applications, systems are inevitably subjected to various interference signals characterized by significant uncertainties in temporal distributions and amplitude intensities. A representative example includes outlier measurements generated by low-reliability sensors. Under such complex operating conditions, the conventional assumption of Gaussian-distributed process and measurement noise often fails, as system noise typically exhibits non-Gaussian heavy-tailed statistical properties (e.g., Gaussian mixture or Student’s t-distributions). These characteristics disrupt the optimality conditions of CKF, leading to substantial degradation in state estimation accuracy and filter stability, thereby severely limiting CKF practical applicability in non-Gaussian noise environments.

To address the challenges posed by unknown disturbances and non-Gaussian heavy-tailed noise in dynamic systems, numerous robust filtering algorithms have been proposed in recent years. Among these, Kalman filter variants based on MCC have emerged as a research focus due to their exceptional disturbance rejection capabilities. In contrast to the traditional MMSE criterion, which relies solely on second-order moments of the innovation, the MCC framework enhances robustness against the abnormal disturbances by jointly exploiting both second- and higher-order moment information of the innovation. Consequently, integrating MCC into the CKF framework yields a robust hybrid algorithm—MCC-CKF. This algorithm establishes a cooperative optimization mechanism between the error covariance matrix and the kernel function, effectively improving state estimation accuracy and filtering stability.

### 2.3. Principle of MCC-CKF

In the framework of a nonlinear dynamic system model, the optimization objective function based on MCC is formulated as follows:

(16) $$J_{MCC}(x_{k})=G_{\sigma_{k}}\left({\left\|y_{k}-h(x_{k})\right\|}_{R_{k}^{-1}}\right)+G_{\sigma_{k}}\left({\left\|x_{k}-f(x_{k-1})\right\|}_{P_{k|k-1}^{-1}}\right),$$

where $\left||\cdot |{|}_{D}^{2}={\left(\cdot \right)}^{T}D^{-1}(\cdot \right)$ denotes the squared Mahalanobis distance of the covariance matrix D, representing the standardized discrepancy under the covariance structure.

To address the limitation that a traditional Gaussian kernel function is not sensitive to capturing feature correlations, a Gaussian kernel function based on a square Mahalanobis distance is reconstructed as follows:

(17) $$G_{\sigma_{k}}\left(||\cdot |{|}_{D}\right)=\exp\left(-\frac{||\cdot |{|}_{D}^{2}}{2\sigma_{k}^{2}}\right).$$

The optimal estimate $x_{k}$ is obtained by maximizing $J_{\mathrm{MCC}}\left(x_{k}\right)$,

(18) $${\hat{x}}_{k}=\underset{x_{k}}{\mathrm{argmax}}\;J_{\mathrm{MCC}}\left(x_{k}\right).$$

Consequently, the state estimate of the original CKF in (9) is rewritten as

(19) $${\hat{x}}_{k}={\hat{x}}_{k|k-1}+K_{k}^{*}\left(y_{k}-{\hat{y}}_{k|k-1}\right),$$

where $K_{k}^{*}$ is defined as follows:

(20) $$K_{k}^{*}=L_{k}P_{xy,k}{\left(P_{yy,k}^{*}\right)}^{-1}.$$

Here, $L_{k}$ serves as an additional adjustment factor to suppress the influence of anomalous disturbances and iteratively refine the estimation accuracy of ${\hat{x}}_{k}$.

(21) $$L_{k}=\exp\left(-\frac{{\left\|y_{k}-h\left(X_{i,k|k-1}\right)\right\|}_{R_{k}}}{2\sigma_{k}^{2}}\right).$$

The modified measurement covariance matrix is

(22) $$P_{yy,k}^{*}=L_{k}\frac{1}{2n}\sum_{i=1}^{2n}\left(Y_{i,k|k-1}-{\hat{y}}_{k|k-1}\right){\left(Y_{i,k|k-1}-{\hat{y}}_{k|k-1}\right)}^{T}+R_{k}.$$

Accordingly, the covariance update is modified to

(23) $$P_{k}=P_{k|k-1}-L_{k}P_{xy,k}\left(P_{yy,k}^{*}\right)P_{xy,k}^{T}.$$

The complete MCC-CKF (Algorithm 1) is summarized as follows.

**Algorithm 1:** MCC-CKFInput: ${\hat{x}}_{k-1},\;P_{k-1}$(1) Prediction:Generate 2n symmetric cubature points in (2), and calculate the predicted state in (3) and the corresponding covariance in (4).(2) Update:Predict measurements in (5);Initialize m=0, and set ${\hat{x}}_{m,k}={\hat{x}}_{k|k-1}$, $P_{m,k}=P_{k|k-1}$ and ξ as the iteration threshold.Iterate:The adjustment factor $L_{m,k}$ in (21), the measurement covariance $P_{yy,m,k}^{*}$ in (22), the cross-covariance $P_{xy,k}$ in (7), and the Kalman gain $K_{m,k}^{*}$ in (20) are calculated successively.Refine the state estimate ${\hat{x}}_{m+1,k}$ in (19) and the covariance $P_{m,k}$ in (23).Until: $\left(\left\|{\hat{x}}_{m+1,k}-{\hat{x}}_{m,k}\right\|/\left\|{\hat{x}}_{m,k}\right\|\leq \xi \right)$;Return ${\hat{x}}_{k}={\hat{x}}_{M,k}$ and $P_{k}=P_{M,k}$, where M is iterations.Output: ${\hat{x}}_{k}$, $P_{k}$.

### 2.4. Limitation and Improvement of MCC-CKF

As illustrated in Algorithm 1, MCC-CKF extends CKF to non-Gaussian environments by adaptively weighting the innovation covariance through a kernel function and iteratively optimizing the Kalman gain and covariance matrices using the adjustment factor $L_{k}$. This mechanism effectively suppresses the influence of large estimation errors, significantly enhancing robustness under non-Gaussian noise. However, this improvement incurs higher computational complexity due to kernel operations, iterative optimization, and parameter adjustment—particularly, the selection of the kernel size $\sigma_{k}$. As a critical hyperparameter, $\sigma_{k}$ directly governs the computation of $L_{k}$ and propagates its sensitivity to external disturbances to the gain matrix by means of $L_{k}$. In (21), a smaller $\sigma_{k}$ reduces $L_{k}$, leading to diminished gain magnitudes $K_{k}^{*}$ in (20) and potential performance degradation or even divergence under Gaussian noise. Conversely, if $\sigma_{k}\to \infty$, $L_{k}\to 1$, then $K_{k}^{*}\to K_{k}$ and $P_{yy,k}^{*}\to P_{yy,k}$, and MCC-CKF degenerates into the standard CKF. Thus, the rational selection of kernel size is pivotal to balancing estimation accuracy and robustness in MCC-CKF.

Given the non-stationary nature of external disturbances in temporal distribution and amplitude, the optimal kernel size varies with time-varying disturbance environments. Therefore, it is necessary to adopt the kernel scale adaptive adjustment strategy based on real-time data to make the adjustment factor dynamically respond to disturbance variations. This mechanism endows the filter with real-time responsiveness to unknown time-varying disturbances, achieving a balanced optimization between disturbance rejection and estimation accuracy through parameter coupling. Existing approaches, such as the adaptive method in [25], dynamically adjust the kernel size based on the weighted norm of measurement residuals at each iteration ($\sigma_{k}={\left\|y_{k}-H_{k}{\hat{x}}_{k|k-1}\right\|}_{R_{k}^{-1}}$). While this method improves robustness against non-Gaussian noise compared to filters relying on default or heuristic kernel selections [20,26,27], integrating the adjustment factor $L_{k}$ with a residual-dependent adaptation of the kernel size constrains $L_{k}$ to a static value, thereby preventing real-time responsiveness to varying disturbance intensities. Consequently, this adaptive strategy remains insufficient to counteract unknown disturbances of varying intensities. Therefore, kernel size adjustment transitions from static or heuristic approaches to data-driven probabilistic inference, substantially enhancing the filters’ robustness in non-stationary non-Gaussian environments.

To eliminate reliance on heuristic parameter tuning, this paper proposes the VBMCC-CKF framework, which recursively estimates the system state while dynamically adapting the kernel size via VB theory [33]. Leveraging VB theory, the joint posterior distribution of the state and kernel size is approximated using factorized distributions (Gaussian and inverse gamma), ensuring computational tractability and efficiency. Integrated with the state estimation framework of MCC-CKF, the proposed method achieves real-time adaptability to unknown time-varying disturbances and significantly improves robustness in non-stationary non-Gaussian environments.

## 3. VBMCC-CKF with Adaptive Kernel Size Adjustment Mechanism

### 3.1. Kernel Size Modeling and Joint Estimation of State and Kernel Size

Let the Gaussian kernel size and the state $x_{k}$ be modeled as random variables and define $\varphi_{k}≜\sigma_{k}^{2}$. Leveraging the conjugacy of the Gaussian-inverse gamma distribution, which ensures closed-form posterior updates and computational efficiency, the kernel size $\varphi_{k}$ is modeled as a random variable following an inverse gamma distribution:

(24) $$\varphi_{k}∼\mathrm{IG}(\varphi_{k};\alpha_{k},\beta_{k}).$$

The state variables follow a Gaussian distribution:

(25) $$x_{k}∼N(x_{k};{\hat{x}}_{k},P_{k}).$$

To jointly estimate the state variables and kernel size, the joint posterior distribution is formulated as

(26) $$p(x_{k},\varphi_{k}|y_{1:k})=N(x_{k};{\hat{x}}_{k},P_{k})\times \mathrm{IG}(\varphi_{k};\alpha_{k},\beta_{k}),$$

where $p(x_{k},\varphi_{k}|y_{1:k})$ denotes the joint posterior probability density function, and $y_{1:k}$ represents the measurement sequence of the sensor from the start to the current time k. The objective is to estimate the state $x_{k}$ and kernel size $\varphi_{k}$ conditioned on $y_{1:k}$.

### 3.2. Variational Bayesian Iterative Optimization

Because of the coupling of $x_{k}$ and $\varphi_{k}$ in joint posterior distribution, the analytic solution of joint posterior distribution cannot be derived directly by using Bayes’ rule. To decouple the joint posterior, the variational Bayesian approximation [33] is employed to decompose the joint posteriori distribution into two factorized independent distributions $q_{x}(x_{k})$ and $q_{\varphi }(\varphi_{k})$.

(27) $$p(x_{k},\varphi_{k}|y_{1:k})\approx q_{x}(x_{k})q_{\varphi }(\varphi_{k}).$$

The Kullback–Leibler (KL) divergence quantifies the discrepancy between two probability distributions. Within the variational Bayesian framework, the goal is to minimize the KL divergence between the factorized approximation $q_{x}(x_{k})q_{\varphi }(\varphi_{k})$ and the true joint posterior $p(x_{k},\varphi_{k}|y_{1:k})$, expressed as follows:

(28) $$\mathrm{KL}(q_{x}(x_{k})q_{\varphi }(\varphi_{k})||p(x_{k},\varphi_{k}|y_{1:k}))=\int q_{x}q_{\varphi }\log\frac{q_{x}q_{\varphi }}{p(x_{k},\varphi_{k}|y_{1:k})}dx_{k}d\varphi_{k}.$$

Optimizing towards minimal KL divergence inherently ensures the evidence lower bound (ELBO) reaches its theoretical maximum [39], thereby identifying optimal $q_{x}(x_{k})$ and $q_{\varphi }(\varphi_{k})$. The variational Bayesian iterative optimization realizes the online adaptive selection of kernel size by alternating between updating $q_{x}(x_{k})$ and $q_{\varphi }(\varphi_{k})$ and progressively approximating the true posterior distribution. Its core value is to transform the complex joint estimation problem into a subproblem that can be solved analytically, that is, the alternating optimization calculation of two independent distributions, while ensuring the real-time and anti-interference ability of the algorithm. The alternating optimization rule is as follows:

(1) Fix the kernel size $\varphi_{k}$ and update the state distribution $q_{x}(x_{k})$: the following update rule is derived analytically by minimizing $\mathrm{KL}(q_{x}(x_{k})||p(x_{k}|\varphi_{k},y_{1:k}))$.
  (29) $$q_{x}(x_{k})\propto \exp(E_{q_{\varphi }}[\log p(y_{k},x_{k},\varphi_{k}|y_{1:k-1})]).$$
(2) Fix the state $x_{k}$ and update the kernel size distribution $q_{\varphi }(\varphi_{k})$: the following update rule is derived analytically by minimizing $\mathrm{KL}(q_{\varphi }(\varphi_{k})||p(\varphi_{k}|x_{k},y_{1:k}))$.
  (30) $$q_{\varphi }(\varphi_{k})\propto \exp(E_{q_{x}}[\log p(y_{k},x_{k},\varphi_{k}|y_{1:k-1})]).$$

**Remark** **1.**  *The conjugacy between the Gaussian distribution (state) and the inverse gamma distribution (kernel size) guarantees the closed-form solution of the posterior distribution. The updated formulas of state and kernel size are derived by minimizing the KL divergence. All updated formulas are directly derived by analytic expectation calculation, requiring only 2–3 iterations for convergence. This avoids Monte Carlo integration or numerical optimization, satisfying real-time computational demands.*

### 3.3. Closed-Form Derivation

The closed-form derivation hinges on leveraging the conjugacy between the Gaussian and inverse gamma distributions within the variational Bayesian framework, enabling analytical updates of the joint posterior distribution for the state $x_{k}$ and kernel size $\varphi_{k}$. The detailed derivation proceeds as follows:

**(i) Initialization:** To facilitate recursive computation, it is assumed that the prior distributions of the state and the kernel size at time k−1 also satisfy the conjugation condition, i.e., $x_{k-1}∼N(x_{k-1};{\hat{x}}_{k-1},P_{k-1})$ and $\varphi_{k-1}∼\mathrm{IG}(\varphi_{k-1};\alpha_{k-1},\beta_{k-1})$. The joint posterior probability density function in (31) is approximated as the product of the Gaussian distribution and the inverse gamma distribution.

(31) $$p(x_{k-1},\varphi_{k-1}|y_{1:k-1})=p(x_{k-1}|y_{1:k-1})p(\varphi_{k-1}|y_{1:k-1})\approx N(x_{k-1};{\hat{x}}_{k-1},P_{k-1})\mathrm{IG}(\varphi_{k-1};\alpha_{k-1},\beta_{k-1}).$$

**(ii) Prediction Step:** To compute the joint predicted probability density function $p(x_{k},\varphi_{k}|y_{1:k-1})$ at time k, a dynamic evolution model $p(\varphi_{k}|\varphi_{k-1})$ is formulated for the kernel size $\varphi_{k}$. The kernel size parameters propagate temporally through a heuristic model, which embodies an exponentially decaying memory mechanism. This mechanism ensures that historical information diminishes gradually over time, thereby enhancing adaptability to dynamically disturbed environments.

(32) $$\left\{\begin{array}{l} \alpha_{k|k-1}=\mu \alpha_{k-1|k-1}\\ \beta_{k|k-1}=\mu \beta_{k-1|k-1}, \end{array}\right.$$

where μ∈(0,1] governs the time-varying behavior of the kernel size, referred to as the decay factor. Smaller values of μ accelerate the temporal adaptation of the kernel size, enabling rapid response to dynamic disturbed conditions. 

Given the absence of physical correlation between the state variables and kernel size, their dynamic evolution models are postulated to be mutually independent:

(33) $$p(x_{k},\varphi_{k}|x_{k-1},\varphi_{k-1})=p(x_{k}|x_{k-1})p(\varphi_{k}|\varphi_{k-1})$$

Further, leveraging the Chapman–Kolmogorov equation [33], the independence of the predicted probability density functions $p\left(x_{k}|y_{1:k-1}\right)$ and $p\left(\varphi_{k}|y_{1:k-1}\right)$ is established, leading to the following:

(34) $$p\left(x_{k},\varphi_{k}|y_{1:k-1}\right)=p\left(x_{k}|y_{1:k-1}\right)p\left(\varphi_{k}|y_{1:k-1}\right).$$

Given the propagation characteristics of Gaussian noise, the initial state $x_{k-1}∼N(x_{k-1};{\hat{x}}_{k-1},P_{k-1})$ undergoes deterministic sampling (e.g., cubature points in (2)) for the nonlinear state transition function f(·) in (1). By integrating the predicted state mean in (3), predicted covariance in (4), and Equations (31) and (34), the state prediction distribution remains approximately Gaussian: $p\left(x_{k}|y_{1:k-1}\right)\approx N(x_{k};{\hat{x}}_{k|k-1},P_{k|k-1})$. Similarly, leveraging the dynamic evolution model for the kernel size in (32), the predicted distribution of kernel size retains its inverse gamma form: $p(\varphi_{k}|y_{1:k-1})=\mathrm{IG}(\varphi_{k};\alpha_{k|k-1},\beta_{k|k-1})$. Therefore, substituting $p\left(x_{k}|y_{1:k-1}\right)$ and $p(\varphi_{k}|y_{1:k-1})$ into Equation (34), the joint predictive probability density function $p\left(x_{k},\varphi_{k}|y_{1:k-1}\right)$ is calculated as follows:

(35) $$p\left(x_{k},\varphi_{k}|y_{1:k-1}\right)\approx N(x_{k};{\hat{x}}_{k|k-1},P_{k|k-1})\mathrm{IG}(\varphi_{k};\alpha_{k|k-1},\beta_{k|k-1}).$$

**(iii) Update:** The joint posterior distribution of the state $x_{k}$ and kernel size $\varphi_{k}$ is analytically updated via the variational Bayesian iterative optimization outlined in Section 3.2, resolving the coupling between $x_{k}$ and $\varphi_{k}$. By minimizing the KL divergence in (28), the factorized distributions $q_{x}(x_{k})$ and $q_{\varphi }(\varphi_{k})$ are alternately optimized.

(1) Update state distribution $q_{x}(x_{k})$ (fix $\varphi_{k}$): Integrating the predictive probability density function in (34), the joint probability $p\left(y_{k},x_{k},\varphi_{k}|y_{1:k-1}\right)$ can be decomposed as follows:

(36) $$\begin{array}{l} p\left(y_{k},x_{k},\varphi_{k}|y_{1:k-1}\right)=p\left(y_{k}|x_{k},\varphi_{k}\right)p\left(x_{k},\varphi_{k}|y_{1:k-1}\right)\\ \;\;\;\;\;\;\;\;\;\;\;\;=p\left(y_{k}|x_{k},\varphi_{k}\right)p\left(x_{k}|y_{1:k-1}\right)p\left(\varphi_{k}|y_{1:k-1}\right), \end{array}$$

where $p\left(y_{k}|x_{k},\varphi_{k}\right)$ represents the joint likelihood function. This likelihood is defined by the Gaussian kernel in (37), which incorporates second- and higher-order moment information of the innovation to mitigate the influence of disturbances on the dynamic system:

(37) $$\begin{array}{l} p(y_{k}|x_{k},\varphi_{k})=G_{\varphi_{k}}\left(||y_{k}-h(x_{k})|{|}_{R_{k}^{-1}}\right)\\ \;\;\;\;\;=\frac{1}{{\left(2\pi \varphi_{k}\right)}^{d/2}}\exp\left(-\frac{{\left(y_{k}-h\left(x_{k}\right)\right)}^{T}R_{k}^{-1}\left(y_{k}-h\left(x_{k}\right)\right)}{2\varphi_{k}}\right), \end{array}$$

where d denotes the measurement dimension. The variational update proceeds by integrating Equations (35) and (37) and computing the expectation $E_{q_{\varphi }}[\cdot ]$ of $\log p\left(y_{k},x_{k},\varphi_{k}|y_{1:k-1}\right)$ with respect to $q_{\varphi }(\varphi_{k})$, yielding the following:

(38) $$\begin{array}{l} q_{x}(x_{k})\propto \exp\left(-\frac{1}{2}{\left(x_{k}-{\hat{x}}_{k|k-1}\right)}^{T}P_{k|k-1}^{-1}\left(x_{k}-{\hat{x}}_{k|k-1}\right)\right)\\ \;\;\;\;\cdot \exp\left\{-\frac{1}{2}E_{q_{\varphi }}\left[\frac{{\left(y_{k}-h\left(x_{k}\right)\right)}^{T}R_{k}^{-1}\left(y_{k}-h\left(x_{k}\right)\right)}{\varphi_{k}}\right]\right\}. \end{array}$$

Consequently, $q_{x}(x_{k})$ retains a Gaussian form as the product of two Gaussian distributions, with the measurement noise covariance scaled by the following:

(39) $$q_{x}\left(x_{k}\right)\propto N(y_{k};h(x_{k}),\varphi_{k}R_{k})N(x_{k};{\hat{x}}_{k|k-1},P_{k|k-1}).$$

For $q_{x}(x_{k})=N(x_{k};{\hat{x}}_{k},{\tilde{P}}_{k})$, the state estimate ${\hat{x}}_{k}$ and covariance ${\tilde{P}}_{k}$ are updated as follows:

(40) $${\hat{x}}_{k}={\hat{x}}_{k|k-1}+{\tilde{K}}_{k}\left(y_{k}-{\hat{y}}_{k|k-1}\right),$$

(41) $${\tilde{P}}_{k}={\tilde{P}}_{k|k-1}-{\tilde{P}}_{xy,k}\left({\tilde{P}}_{yy,k}\right){\tilde{P}}_{xy,k}^{T}={\tilde{P}}_{k|k-1}-{\tilde{K}}_{k}{\tilde{P}}_{yy,k}{\left({\tilde{K}}_{k}\right)}^{T},$$

where ${\tilde{K}}_{k}$ is defined as follows:

(42) $${\tilde{K}}_{k}={\tilde{P}}_{xy,k}{\left({\tilde{P}}_{yy,k}\right)}^{-1}.$$

The measurement covariance matrix is

(43) $${\tilde{P}}_{yy,k}=\frac{1}{2n}\sum_{i=1}^{2n}\left(Y_{i,k|k-1}-{\hat{y}}_{k|k-1}\right){\left(Y_{i,k|k-1}-{\hat{y}}_{k|k-1}\right)}^{T}+\varphi_{k}R_{k}.$$

The cross-covariance matrix ${\tilde{P}}_{xy,k}$ is consistent with Equation (7):

(44) $${\tilde{P}}_{xy,k}=\frac{1}{2n}\sum_{i=1}^{2n}\left(\chi_{i,k|k-1}-{\hat{x}}_{k|k-1}\right){\left(Y_{i,k|k-1}-{\hat{y}}_{k|k-1}\right)}^{T}.$$

(2) Update kernel size distribution $q_{\varphi }(\varphi_{k})$ (fix $x_{k}$):

The variational update proceeds by computing the expectation $E_{q_{x}}[\cdot ]$ of $\log p\left(y_{k},x_{k},\varphi_{k}|y_{1:k-1}\right)$ with respect to $q_{x}(x_{k})$ and yielding the following:

(45) $$\begin{array}{cc} q_{\varphi }(\varphi_{k}) & \propto \exp\left(-\alpha_{k|k-1}-\frac{d}{2}-1\right)\varphi_{k}\\ & \cdot \exp\left\{\frac{-\beta_{k|k-1}-\frac{1}{2}E_{q_{x}}\left[{\left(y_{k}-h\left(x_{k}\right)\right)}^{T}R_{k}^{-1}\left(y_{k}-h\left(x_{k}\right)\right)\right]}{\varphi_{k}}\right\}. \end{array}$$

Therefore, $q_{\varphi }(\varphi_{k})$ retains its inverse gamma distribution. By applying the linearization approximation $h\left(x_{k}\right)\approx h\left({\hat{x}}_{k}\right)$, the residual $\varepsilon_{k}=y_{k}-h\left({\hat{x}}_{k}\right)$ yields the following closed-form update for the kernel size parameter:

(46) $$\alpha_{k}=\alpha_{k|k-1}+\frac{d}{2},$$

(47) $$\beta_{k}=\beta_{k|k-1}+\frac{1}{2}{\varepsilon_{k}}^{T}R_{k}^{-1}\varepsilon_{k}.$$

The expectation of the kernel size is $E[\varphi_{k}]=\beta_{k}/\left(\alpha_{k}-1\right)$.

**Remark** **2.**  *For online adaptability of the kernel size, $\varphi_{k}$ dynamically adjusts based on the residual $\varepsilon_{k}$. When $\varepsilon_{k}$ increases (indicating the presence of outliers), $\beta_{k}$ increases, thereby enlarging $\varphi_{k}$ to reduce the weighting of current measurements (suppressing disturbance). Conversely, when $\varepsilon_{k}$ decreases, $\varphi_{k}$ diminishes, enhancing the utilization of measurement information. This mechanism ensures adaptive adjustment of the kernel size parameter in response to varying disturbance intensities.*

Through the aforementioned derivations, the joint posterior probability density function at time k can be approximated as the product of a Gaussian distribution and an inverse gamma distribution after alternating optimization. The parameters of the Gaussian and inverse gamma distributions are iteratively determined via Equations (40)–(44) and (46)–(47), respectively.

(48) $$p(x_{k},\varphi_{k}|y_{1:k})\approx N(x_{k};{\hat{x}}_{k},{\tilde{P}}_{k})\mathrm{IG}(\varphi_{k};\alpha_{k},\beta_{k}).$$

The comprehensive process of the proposed VBMCC-CKF method is outlined below (Algorithm 2), integrating the alternating optimization framework and closed-form parameter updates derived above.

**Algorithm 2:** VBMCC-CKFInput: $\left({\hat{x}}_{k-1},P_{k-1},\alpha_{k-1},\beta_{k-1}\right)$
(1)Prediction:
a. Generate 2n symmetric cubature points (Equation (2)) for state propagation (Equation (3)) and covariance matrix computation (Equation (4)).b. Obtain $\alpha_{k|k-1}$ and $\beta_{k|k-1}$ through the dynamic evolution model defined in (32).c. Compute the joint predictive probability density function $p(x_{k},\varphi_{k}|y_{1:k-1})$ using Equation (35).
(2)Update:
a. Propagate the predicted cubature points through the measurement function h(·) to generate measurement cubature points: $Y_{i,k|k-1}=h\left(X_{i,k|k-1}\right)$, i=1,…,2n. Predict the measurement mean ${\hat{y}}_{k|k-1}$ via Equation (5).b. Initialize m=0 with ${\hat{x}}_{m,k}={\hat{x}}_{k|k-1}$, ${\tilde{P}}_{m,k}=P_{k|k-1}$, $\alpha_{k}=\alpha_{k|k-1}+\frac{d}{2}$, and $\beta_{m,k}=\beta_{k|k-1}$. Subsequently, execute the variational Bayesian iterative optimization as follows:(1) Compute the kernel size $\varphi_{i,k}=\beta_{i,k}/\left(\alpha_{k}-1\right)$.(2) Calculate the measurement covariance matrix ${\tilde{P}}_{yy,m,k}$ using Equation (43)${\tilde{P}}_{yy,m+1,k}=\frac{1}{2n}\sum_{i=1}^{2n}\left(Y_{i,k|k-1}-{\hat{y}}_{k|k-1}\right){\left(Y_{i,k|k-1}-{\hat{y}}_{k|k-1}\right)}^{T}+\varphi_{m,k}R_{k}$.(3) Compute the cross-covariance matrix ${\tilde{P}}_{xy,k}$ using Equation (44)${\tilde{P}}_{xy,k}=\frac{1}{2n}\sum_{i=1}^{2n}\left(\chi_{i,k|k-1}-{\hat{x}}_{k|k-1}\right){\left(Y_{i,k|k-1}-{\hat{y}}_{k|k-1}\right)}^{T}$.(4) Compute the Kalman gain matrix using Equation (42): ${\tilde{K}}_{m+1,k}={\tilde{P}}_{xy,k}{\left({\tilde{P}}_{yy,m+1,k}\right)}^{-1}$.(5) Update the state estimate ${\hat{x}}_{m+1,k}$ and covariance matrix $P_{m+1,k}$ via Equations (40) and (41):${\hat{x}}_{m+1,k}={\hat{x}}_{k|k-1}+{\tilde{K}}_{m+1,k}\left(y_{k}-{\hat{y}}_{k|k-1}\right),\;P_{m+1,k}=P_{k|k-1}-{\tilde{P}}_{xy,k}\left({\tilde{P}}_{yy,m+1,k}\right){\tilde{P}}_{xy,k}^{T}$.(6) Update the kernel size parameters: $\beta_{m,k}=\beta_{k|k-1}+\frac{1}{2}{\varepsilon_{k}}^{T}R_{k}^{-1}\varepsilon_{k}$, where $\varepsilon_{k}=y_{k}-h\left({\hat{x}}_{m+1,k}\right)$.(7) Iteration termination criterion: If $\left\|{\hat{x}}_{m+1,k}-{\hat{x}}_{m,k}\right\|/\|{\hat{x}}_{m,k}\|>\xi$, return to Step (1) to continue iteration. Otherwise, set ${\hat{x}}_{k}={\hat{x}}_{M,k}$, ${\tilde{P}}_{k}={\tilde{P}}_{M,k}$, $\;\alpha_{k}=\alpha_{k|k-1}$, and $\beta_{k}=\beta_{M,k}$, where M denotes the total number of iterations. Finally, compute the joint posterior probability density function $p(x_{k},\varphi_{k}|y_{1:k})\approx N(x_{k};{\hat{x}}_{k},{\tilde{P}}_{k})\mathrm{IG}(\varphi_{k};\alpha_{k},\beta_{k})$ and return $p(x_{k},\varphi_{k}|y_{1:k})$.Output: ${\hat{x}}_{k},{\tilde{P}}_{k},\alpha_{k},\beta_{k}$.

## 4. Analysis of VBMCC-CKF

Section 3.3 rigorously derives the closed-form solution for the joint posterior distribution of the state and kernel size in VBMCC-CKF using variational Bayesian methods. The derived joint posterior retains an approximate factorization into Gaussian and inverse gamma distributions after iterative optimization. This section first supplements the mathematical validation with a focus on the closed-form kernel size update. Subsequently, anti-disturbance mechanism analyses are conducted to evaluate the robustness of the kernel size adaptation. Finally, the computational complexity of VBMCC-CKF is analyzed to assess its practical feasibility.

### 4.1. Mathematical Verification Using Kernel Size Closed-Form Update as an Example

Assuming the prior distribution of the kernel size as $p(\varphi_{k-1}|y_{1:k-1})=\mathrm{IG}(\varphi_{k-1};\alpha_{k-1|k-1},\beta_{k-1|k-1})$, the predictive distribution is obtained by the dynamic evolution model propagation in (32):

(49) $$p(\varphi_{k}|y_{1:k-1})=\mathrm{IG}(\varphi_{k};\alpha_{k|k-1},\beta_{k|k-1})$$

The measurement likelihood function is defined as follows:

(50) $$p\left(y_{k}|\varphi_{k}\right)\propto \varphi_{k}^{-d/2}\exp\left(-\frac{\varepsilon_{k}^{T}R_{k}^{-1}\varepsilon_{k}}{2\varphi_{k}}\right)$$

According to Bayes’ theorem, the posterior distribution of the kernel size is proportional to the product of the likelihood and prior:

(51) $$p\left(\varphi_{k}|y_{1:k}\right)\propto p\left(y_{k}|\varphi_{k}\right)p\left(\varphi_{k}|y_{1:k-1}\right)$$

Substituting the specific forms of Equations (49) and (50) into Equation (51) yields the following:

(52) $$p\left(\varphi_{k}|y_{1:k}\right)\propto \varphi_{k}^{-\alpha_{k|k-1}-d/2-1}\exp\left(-\frac{\beta_{k|k-1}+\varepsilon_{k}^{T}R_{k}^{-1}\varepsilon_{k}/2}{\varphi_{k}}\right)$$

The posterior distribution retains the inverse gamma form $\mathrm{IG}(\varphi_{k};\alpha_{k|k},\beta_{k|k})$, thereby validating the correctness of the closed-form update mechanism.

### 4.2. Anti-Disturbance Mechanism Analysis

A steady state implies that the system state and parameters no longer undergo significant changes over time, meaning the parameters converge to either a fixed value or exhibit periodic variation. For kernel size parameters, steady state analysis requires determining the long-term behavior of the kernel scale parameters and whether the expected value converges with it. The kernel size parameters are dynamically updated through an adaptive adjustment mechanism governed by Equations (46) and (47) and the model in (32). Under the condition that the residual $\varepsilon_{k}$ exhibits bounded expectation (indicating system stability), if the covariance of $\varepsilon_{k}$ tends to stabilize, then the expected value of $\varepsilon_{k}$ satisfies $E[{\varepsilon_{k}}^{T}R_{k}^{-1}\varepsilon_{k}]=\mathrm{tr}(R_{k}^{-1}S)$, where S is the steady-state covariance matrix of $\varepsilon_{k}$. Furthermore, based on the model in (32) and the update formulas in (46) and (47) of $\alpha_{k}$ and $\beta_{k}$, the parameters $\alpha_{k}$ and $\beta_{k}$ demonstrate a linear growth trend over time, asymptotically approaching stable values, which are denoted as $\alpha_{\infty }$ and $\beta_{\infty }$. Consequently, the expectation of the kernel size converges to a steady-state value $E[\varphi_{k}]=\beta_{\infty }/\left(\alpha_{\infty }-1\right)=\mathrm{tr}(R_{k}^{-1}S)/d-2(1-\mu )$, thereby ensuring the stability of the gain matrix. In scenarios where outliers cause abrupt increases in $\varepsilon_{k}$, $\beta_{k}$ rapidly increases, triggering an adaptive adjustment of $\varphi_{k}$ to reduce the gain matrix and suppress outlier interference effectively. Subsequently, due to the effect of the decay factor μ in smoothing historical information, it avoids over-suppressing normal data and gradually returns to the steady state.

Steady-state analysis validates the long-term stability and reliability of VBMCC-CKF, ensuring robust estimation performance in diverse adversarial environments. Notably, compared to the MCC-CKF in Algorithm 1, the gain matrix is reformulated by integrating Equations (7), (20) and (22), as follows:

(53) $$K_{k}^{*}=P_{xy,k}{\left(\frac{1}{2n}\sum_{i=1}^{2n}\left(Y_{i,k|k-1}-{\hat{y}}_{k|k-1}\right){\left(Y_{i,k|k-1}-{\hat{y}}_{k|k-1}\right)}^{T}+\frac{1}{L_{k}}R_{k}\right)}^{-1}.$$

Similarly, by integrating Equations (42)–(44), the gain matrix of VBMCC-CKF is reformulated as follows:

(54) $${\tilde{K}}_{k}=P_{xy,k}{\left(\frac{1}{2n}\sum_{i=1}^{2n}\left(Y_{i,k|k-1}-{\hat{y}}_{k|k-1}\right){\left(Y_{i,k|k-1}-{\hat{y}}_{k|k-1}\right)}^{T}+\varphi_{k}R_{k}\right)}^{-1}.$$

A comparative analysis of $K_{k}^{*}$ and ${\tilde{K}}_{k}$ reveals that VBMCC-CKF and MCC-CKF are structurally equivalent. Both the reciprocal of the adjustment factor ($1/L_{k}$) and the kernel size ($\varphi_{k}$) serve to modulate $R_{k}$ in response to disturbances. Nevertheless, regardless of whether the kernel size is selected based on default values, empirical rules, or adaptive adjustment, MCC-CKF exhibits limited efficacy in mitigating unknown disturbances of varying intensities. Consequently, VBMCC-CKF’s dynamic adaptation of $\varphi_{k}$ in response to disturbance intensity confers superior robustness against unknown time-varying disturbances.

### 4.3. Computational Complexity

The proposed VBMCC-CKF integrates VB methods with CKF while adaptively adjusting the kernel size parameters. Compared to the conventional CKF, VBMCC-CKF incurs a modest increase in computational complexity. Therefore, a concise analysis of its computational complexity is conducted from both time and space complexity perspectives.

In terms of time complexity, the computational complexity of CKF is dominated by the calculation of the Kalman gain, with a time complexity of $O(n^{3})$. VBMCC-CKF introduces variational Bayesian iteration on this basis, requiring multiple iterations in the update step to optimize the state estimation and kernel size parameters. During each iteration, the Kalman gain, related covariance matrices, and kernel size parameters are recalculated. While the scalar operations for updating kernel size parameters introduce minor computational overhead, their impact on the overall complexity is negligible. Consequently, the time complexity per iteration remains $O(n^{3})$ equivalent to the CKF update step. Given that the variational iterations typically converge within 2–3 cycles (denoted as M), the total time complexity of VBMCC-CKF is $O(Mn^{3})$. Although the total computational load increases linearly with M, its complexity is still of the same order as $n^{3}$, and the overall time complexity is controllable.

In terms of space complexity, CKF mainly stores core data, such as cubature points, covariance matrices, and Kalman gain matrices, resulting in a space complexity of $O(n^{2})$. For VBMCC-CKF, additional intermediate variables, such as iteratively updated state estimates and kernel size parameters, must be stored during variational iterations. However, since these variables share the same dimensionality as the core matrices in CKF, no high-dimensional storage overhead is introduced. Thus, the space complexity remains $O(n^{2})$.

This comprehensive analysis demonstrates that VBMCC-CKF achieves significant improvements in robustness and estimation accuracy with only a modest computational overhead, rendering it highly advantageous for addressing challenges in non-Gaussian noise environments. While its time complexity exceeds that of the conventional CKF, the algorithm attains convergence within merely 2–3 iterations through efficient computational steps, thereby satisfying real-time operational requirements and proving well-suited for practical applications demanding real-time performance.

## 5. Simulation Setup and Performance Validation

This section evaluates the estimation performance of the proposed filtering algorithms under two representative scenarios: single-target tracking and multi-target tracking in non-Gaussian noise environments. All simulations were conducted on a hardware platform equipped with AMD Ryzen 7 7840H CPU and 32 GB RAM to ensure computational reproducibility. The bearing-range measurement models and bearing-only model emulate typical radar sensing principles. The noise parameters are configured to reflect real-world non-Gaussian disturbances.

### 5.1. Single-Target Tracking

#### 5.1.1. Benchmark System

The single-target tracking model and sensor model, including the bearing and range, are established. Set a vector $X_{k}={\left[x,y,{\dot{x}}_{k},{\dot{y}}_{k}\right]}^{T}$ that represents the target position and velocity and measurement vector $Y_{k}$ at time k.

(55) $$X_{k}=A(\theta )X_{k-1}+Bw_{k-1},$$

(56) $$Y_{k}=\left[\begin{array}{c} \arctan\left(x_{k}/y_{k}\right)\\ \sqrt{x_{k}^{2}+y_{k}^{2}} \end{array}\right]+v_{k},$$

where $A(\theta )=\left[\begin{array}{cccc} 1 & \frac{\sin(\theta T)}{\theta } & 0 & -\frac{1-\cos(\theta T)}{\theta }\\ 0 & \cos(\theta T) & 0 & -\sin(\theta T)\\ 0 & \frac{1-\cos(\theta T)}{\theta } & 1 & \frac{\sin(\theta T)}{\theta }\\ 0 & \sin(\theta T) & 0 & \cos(\theta T) \end{array}\right]$, and $B=\left[\begin{array}{cc} \frac{T^{2}}{2} & 0\\ T & 0\\ 0 & \frac{T^{2}}{2}\\ 0 & T \end{array}\right]$.

The sampling period is T=0.5 s, and the turn rate is θ=π/75 rad. The process noise and measurement noise are $w_{k-1}$ and $v_{k}$ and $w_{k-1}∼N(0,Q_{k})$, and $Q_{k}=\mathrm{diag}([25,25])$. $v_{k}∼N(0,R_{k}+R_{k,HT})$ is heavy-tailed, $R_{k}=\mathrm{diag}([\delta^{2},\eta^{2}])$, and $R_{k,HT}=\mathrm{diag}([9\delta^{2},50\eta^{2}])$, where δ=π/180 rad and η=10 m are given. Assuming that the initial state $X_{0}$ and its estimates ${\hat{X}}_{0}$ are identical, $X_{0}={\hat{X}}_{0}=[150;0;500;0]$, and the covariance matrix is $P_{0}=\mathrm{diag}([50,50,50,50])$.

#### 5.1.2. Baselines and Metrics for Single-Target Tracking

The comparative methods employed in this simulation comprise five distinct Kalman filter variants: (1) standard CKF; (2) Mahalanobis distance-based adaptive robust CKF (MD-ARCKF); (3) MCC-CKF, utilizing a fixed kernel size (FKSMCC-CKF); (4) empirical MCC-CKF, incorporating experience-based kernel size selection (EKSMCC-CKF); and (5) the novel VBMCC-CKF featuring a kernel size adjustment mechanism based on the variational Bayesian framework. This systematic comparison framework enables comprehensive evaluation of both established techniques and our proposed kernel size adjustment strategy. The convergence threshold is established at ξ=0.01 to govern iteration termination. Within the VBMCC-CKF framework, the hyperparameters governing the kernel size are initialized to $\alpha_{0}=\beta_{0}=3$, representing the prior shape and scale factors, respectively. To ensure rigorous performance assessment, we implemented Monte Carlo simulations comprising N=100 independent realizations, with the root mean square error (RMSE) serving as the statistical evaluation metric. The RMSE formulation is mathematically expressed as

(57) $${\mathrm{RMSE}}_{k}=\frac{1}{N}\sum_{i=1}^{N}\sqrt{{\left(x_{k}^{i}-{\hat{x}}_{k}^{i}\right)}^{2}+{\left(y_{k}^{i}-{\hat{y}}_{k}^{i}\right)}^{2}},$$

where $\left(x_{k}^{i},y_{k}^{i}\right)$ denotes the coordinate of the target at discrete time step k during the i-th Monte Carlo realization (i∈[1,N]), while $\left({\hat{x}}_{k}^{i},{\hat{y}}_{k}^{i}\right)$ represents the corresponding posterior state estimate generated by the filtering algorithm. The average of RMSE (Avg-RMSE) serves as an additional evaluation metric to evaluate the performance over the entire tracking duration, where $\mathrm{Avg}\text{-}\mathrm{RMSE}=\sum_{k=1}^{M_{t}}{\mathrm{RMSE}}_{k}$, and $M_{t}$ is the entire tracking duration.

Two complementary metrics are employed for the performance assessment: (1) the average iterations per time step, the reflecting convergence rate, and (2) the average CPU time, measured via Monte Carlo trials. The former quantifies optimization stability, while the latter benchmarks computational efficiency.

#### 5.1.3. Results and Discussion for Single-Target Tracking

**Figure 1:**
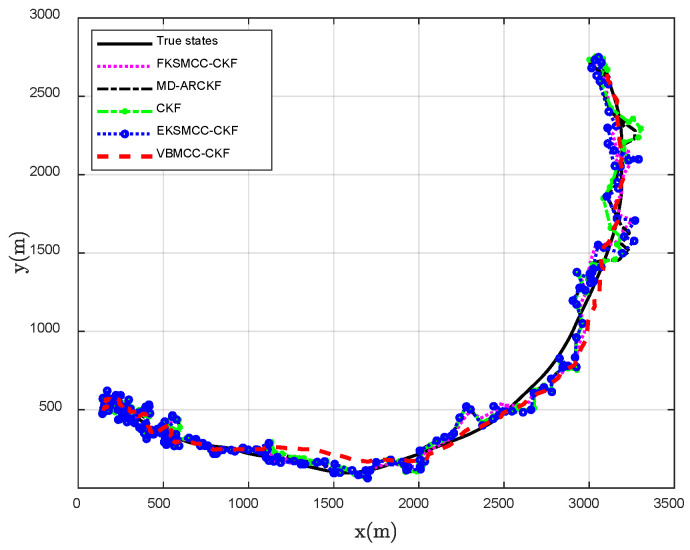
Performance comparison of trajectory estimation in nonlinear target tracking scenarios via single-run Monte Carlo simulations.

**Figure 2:**
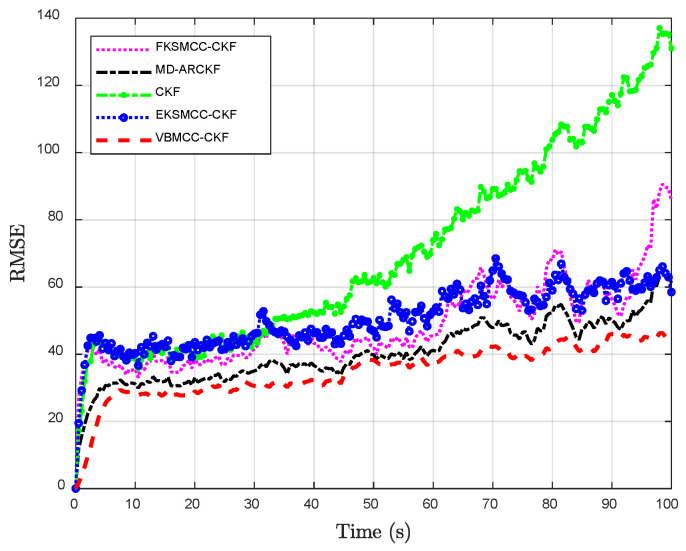
Statistical evaluation of multi-method positioning accuracy: a comparative analysis of RMSE via 100 Monte Carlo simulations.

The proposed VBMCC-CKF demonstrates remarkable advantages in nonlinear target tracking scenarios. As illustrated in Figure 1, the estimated trajectories of VBMCC-CKF exhibit superior alignment with the true motion paths and enhanced smoothness compared to baseline methods such as FKSMCC-CKF, EKSMCC-CKF, MD-ARCKF, and CKF. Quantitative analyses in Figure 2 further validate this superiority: under strong non-Gaussian noise interference, CKF exhibits a peak RMSE fluctuation of 137.0961 m, indicating the worst estimation performance. In contrast, the MCC adopted by the other three methods can more accurately capture the heavy-tail characteristics of large Gaussian noise interference by integrating the higher-order moments of innovation, significantly improving the robustness of tracking estimation. However, the fixed kernel size of FKSMCC-CKF is difficult to adapt to the coupling effects of multi-dimensional measurement variables, resulting in the continuous growth of RMSE. Although the kernel size of EKSMCC-CKF can be adjusted dynamically, it is easy for the adjustment factor to be constant, and the anti-interference effect is limited. The proposed VBMCC-CKF in this paper achieves robust tracking with the lowest RMSE, consistently below 45 m. Specifically, as shown in Table 1, the Avg-RMSE of VBMCC-CKF (34.5919 m) is reduced by 50.87%, 31.65%, and 14.33% compared to FKSMCC-CKF (70.4105 m), EKSMCC-CKF (50.6090 m), and MD-ARCKF (40.3779 m), respectively. This performance enhancement stems from its innovative dynamic kernel size adaptation mechanism, which rapidly responds to varying noise intensities, and the dynamic balance of the kernel size in different dimensions, which realizes the collaborative suppression of noise in different dimensions.

In terms of computational efficiency, Table 1 demonstrates that the proposed VBMCC-CKF achieves an average single-step computation time of 0.0092 s, which aligns with the time complexity of baseline methods, thereby satisfying real-time processing requirements. Notably, while maintaining tracking precision, VBMCC-CKF exhibits an average number of iterations (2.13) comparable to FKSMCC-CKF (1.96) and EKSMCC-CKF (1.99). This efficiency is attributed to its convergence criterion, which terminates 86.43% of iterations within 2.3 steps, ensuring minimal computational overhead without compromising robustness.

**Figure 3:**
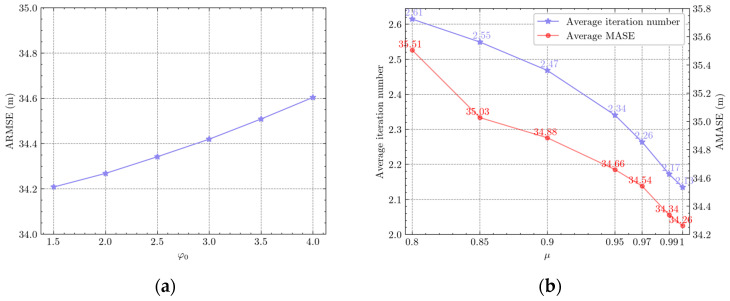
Parametric sensitivity in VBMCC-CKF: (**a**) variation of Avg-RMSE with initial kernel size values ($\varphi_{0}$); (**b**) variation of average iterations and Avg-RMSE with decay factor values (μ).

Furthermore, a sensitivity analysis of two critical parameters—the initial kernel size ($\varphi_{0}$) and the decay factor (μ)—was conducted. As depicted in Figure 3a, the Avg-RMSE exhibits a marginal variation of approximately 0.4 when $\varphi_{0}$ is within the interval [1.5, 4.0], demonstrating that variations in $\varphi_{0}$ within this range exert negligible influence on algorithmic performance, thereby underscoring the robustness of the proposed method. Based on this analysis, $\varphi_{0}$ was ultimately set to 1.5 to optimize computational stability while maintaining high tracking accuracy. As shown in Figure 3b, the increase in the decay factor μ significantly reduces the average iteration count, with its impact exhibiting interval-dependent characteristics. Specifically, when μ∈[0.95,1], the average iteration count decreases rapidly (a reduction of 9%), whereas a smaller reduction (approximately 5%) is observed for μ∈[0.9,0.95]. Correspondingly, although the Avg-RMSE decreases synchronously with the increase in μ, the overall change range is limited. Notably, within μ∈[0.95,1], the Avg-RMSE fluctuates only by approximately 0.4, indicating negligible influence on tracking accuracy. Therefore, selecting μ∈[0.95,1] optimally balances computational efficiency and estimation precision, thereby maximizing algorithmic performance without compromising robustness.

The simulation results demonstrate that under strong non-Gaussian noise interference, the proposed VBMCC-CKF not only enhances tracking estimation accuracy and robustness but also maintains real-time processing capabilities. Furthermore, it provides a novel framework for state estimation in multi-dimensional nonlinear systems by dynamically optimizing kernel parameters to balance measurement credibility across diverse dimensions. This capability holds significant engineering value for applications such as radar systems and multi-sensor fusion architectures.

### 5.2. Multi-Target Tracking

#### 5.2.1. Scenario Construction

In complex dynamic environments, multi-target tracking faces multiple challenges, including bearing-angle measurements, target maneuverability, and non-Gaussian noise characteristics. This simulation simulates the motion of seven targets within a 2000 m × 2000 m surveillance area, observed via a dual-sensor system. The targets follow a variable turn rate model, where θ in the original target model (55) is time-varying:

(58) $$\theta_{k}=\theta_{k-1}+T{\bar{\varpi }}_{k-1},$$

where ${\bar{\varpi }}_{k}$ denotes angular acceleration, the sampling interval is T=1 s, and the augmented state is defined as ${\bar{X}}_{k}={\left[X_{k}^{T},\theta_{k}\right]}^{T}$. The state transition model is formulated as follows:

(59) $$\left[\begin{array}{c} X_{k}\\ \theta_{k} \end{array}\right]=\left[\begin{array}{cc} A\left(\theta_{k-1}\right) & 0_{4\times 1}\\ 0_{1\times 4} & 1 \end{array}\right]\left[\begin{array}{c} X_{k-1}\\ \theta_{k-1} \end{array}\right]+\left[\begin{array}{c} Bw_{k-1}\\ T{\bar{\varpi }}_{k-1} \end{array}\right],$$

where the system noise components $w_{k-1}$ and ${\bar{\varpi }}_{k-1}$ follow independent t-distributions: $w_{k-1}∼T(0,\sum_{w},\eta_{w})$ and ${\bar{\varpi }}_{k-1}∼T(0,\sum_{\bar{\varpi }},\eta_{\bar{\varpi }})$. Here, $\eta_{w}=\eta_{\bar{\varpi }}=4$ represent the degrees of freedom, with scale matrices defined as $\sum_{w}=I_{2}\tau_{w}^{2}\left(\eta_{w}-2\right)/\eta_{w}$ and $\sum_{\bar{\varpi }}=\tau_{\bar{\varpi }}^{2}\left(\eta_{\bar{\varpi }}-2\right)/\eta_{\bar{\varpi }}$, where $\tau_{w}=5\;{m/s}^{2}$ and $\tau_{\bar{\varpi }}=\pi /180\;\mathrm{rad}/s$ govern noise intensity, and $I_{2}$ is the identity matrix. The dual-sensor system employs a bearing-angle measurement mode. Let $\left(x_{S_{i},k},y_{S_{i},k}\right)$ denote the coordinates of the i-th sensor at time k. The measurement equation is defined as follows:

(60) $$Y_{k}=\arctan\left(\left(x_{k}-x_{S_{i},k}\right)/\left(y_{k}-y_{S_{i},k}\right)\right)+v_{k},$$

where the measurement noise $v_{k}$ follows a mixture t-distribution: Under nominal conditions, $v_{k-1}∼T(0,\sum_{v},4)$, where $\sum_{v}=\tau_{v}^{2}\left(\eta_{v}-2\right)/\eta_{v}$, and $\tau_{v}=\pi /1800\;\mathrm{rad}$. At an anomalous time k=25:20:85, the system introduces outliers with equal probability, where the scale matrix switches between $\sum_{v}$ and $10\sum_{v}$.

The motion trajectories of two sensors, $S_{1}$ and $S_{2}$, are configured to follow a constant-velocity curvilinear pattern, with their kinematic parameters defined as follows:

$$\begin{array}{l} S_{1}=\left[-2000+200\sin(t\pi /200);-2000+500\cos(t\pi /200)\right],\\ S_{2}=\left[2000+300\cos(t\pi /200);-1000-500\sin(t\pi /200)\right]. \end{array}$$

The system is configured with a target detection probability $p_{d,k}=0.98$, and the simulation duration is set to 100 s, the clutter model employs a spatial Poisson process with a density of $\lambda =1.25\times 10^{-7}\;m^{-2}$, corresponding to an average of two clutter points per scan.

To address the challenges of multi-target tracking in non-Gaussian noise environments, a validation framework is designed based on Random Finite Set (RFS) theory [40]. The framework constructs a bearing-only measurement scenario to rigorously evaluate complex conditions, such as stochastic target birth/death, clutter interference, and missed detections/false alarms. To circumvent the limitations of traditional data association methods, a multi-Bernoulli filtering framework [36] is employed, enabling unified target set estimation within a probabilistic space. In light of the multi-sensor configuration inherent to this application, a sequential filtering architecture is strategically adopted to ensure scalable sensor data integration and real-time processing feasibility. Three recursive algorithms are comparatively assessed: CKF, EKSMCC-CKF, and VBMCC-CKF. This design effectively isolates the impact of association uncertainties on algorithmic evaluation, highlighting the pivotal role of non-Gaussian noise mitigation mechanisms.

The birth target distributions for EKSMCC-CKF and VBMCC-CKF are defined as a four-component multi-Bernoulli process:

(61) $$\pi_{\Gamma }={\left\{\left[r_{\Gamma ,i},N\left(\bar{X};{\bar{m}}_{\Gamma ,i},{\bar{P}}_{\Gamma }\right)\right]\right\}}_{i=1}^{4},$$

with the following parameter configurations:

(1) Existence probabilities: $r_{\Gamma ,1}=r_{\Gamma ,2}=r_{\Gamma ,3}=r_{\Gamma ,4}=0.02$.
(2) Mean vectors:
  $$\begin{array}{l} {\bar{m}}_{\Gamma ,1}={\left[-1500,0,250,0,0\right]}^{T},\;{\bar{m}}_{\Gamma ,2}={\left[-250,0,1000,0,0\right]}^{T},\\ {\bar{m}}_{\Gamma ,3}={\left[250,0,750,0,0\right]}^{T},\;{\bar{m}}_{\Gamma ,4}={\left[1000,0,1500,0,\right]}^{T}. \end{array}$$
(3) Covariance matrix: ${\bar{P}}_{\Gamma }=\mathrm{diag}{\left({\left[50,50,50,50,\pi /30\right]}^{T}\right)}^{2}$.

The process noise is modeled as follows:

$$\xi_{k}^{x^{*}}=\left[\begin{array}{l} Bw_{k}\\ T{\bar{\varpi }}_{k} \end{array}\right]\sim N\left(0,\left[\begin{array}{cc} B\tau_{w}^{2}I_{2}B^{T} & 0\\ 0 & \tau_{\varpi }^{2} \end{array}\right]\right),$$

where the measurement noise is assumed to follow $v_{k}∼N(0,R)$. To maintain baseline consistency across comparative simulations, CKF excludes the turn rate auxiliary variable ${\bar{\varpi }}_{k}$, while all other parameters remain identical to those of EKSMCC-CKF and VBMCC-CKF. Specifically, the turn rate in the state transition matrix A(θ) is fixed at θ=π/180 rad/s, and the target survival probability is set to $p_{s,k}=0.99$.

#### 5.2.2. Multi-Target Tracking Evaluation Metrics

To quantitatively assess the comprehensive performance of multi-target tracking algorithms, the Optimal Subpattern Assignment (OSPA) distance is adopted as the core metric [36]. This metric provides a multi-dimensional evaluation by jointly quantifying target cardinality estimation errors and state estimation deviations. For a ground-truth target set $Z=\left\{z_{1},\ldots ,z_{m}\right\}$ and an estimated set $\hat{Z}=\left\{{\hat{z}}_{1},\ldots ,{\hat{z}}_{n}\right\}$, the OSPA distance is computed as follows:

(62) $${\bar{d}}_{l}^{c}(X,\hat{X})={\left(\frac{1}{\max(m,n)}\left(\underset{\pi \in \Pi_{\max(m,n)}}{\min}\sum_{i=1}^{\min(m,n)}d^{\left(c\right)}{\left(z_{i},{\hat{z}}_{\pi (i)}\right)}^{l}+c^{l}|m-n|\right)\right)}^{1/l},$$

where $d^{\left(c\right)}\left(z,\hat{z}\right)=\min\left(c,{\left\|z-\hat{z}\right\|}_{2}\right)$ defines the truncated Euclidean distance (with a truncation distance of c=100), l=2 amplifies the penalty for large localization errors, and $\prod_{k}$ denotes the set of permutations of k elements. The truncation mechanism (c) distinguishes localization errors from missed detections/false alarms via cardinality mismatches (|m−n| term), offering a unified benchmark for tracking performance evaluation.

The average OSPA (Avg-OSPA) metric is utilized to quantify multi-target tracking performance over the full observation period, defined as $\mathrm{Avg}\text{-}\mathrm{OSPA}=\sum_{k=1}^{M_{t}}{\mathrm{OSPA}}_{k}$, where $M_{t}$ represents the total tracking duration.

Furthermore, two supplementary metrics are introduced for multi-target tracking performance evaluation: the Hit Rate (HR), defined as the percentage of estimated positions deviating less than 40 m from their true positions, and the RMSE of tracked points (RMSE-TP), which quantifies localization accuracy exclusively for trajectory segments where tracking is sustained. These metrics collectively enhance the granularity of performance assessment by distinguishing between tracking continuity (via HRs) and precision in successfully maintained tracks (via RMSE-TP).

#### 5.2.3. Results and Discussion for Multi-Target Tracking

**Figure 4:**
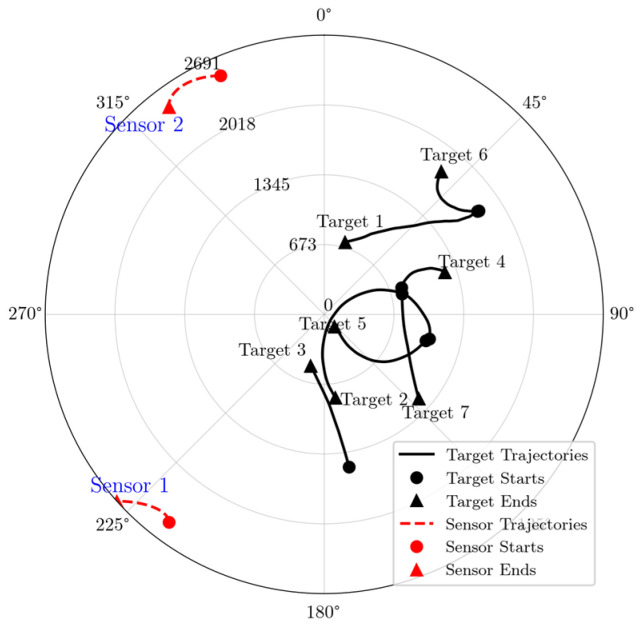
The motion trajectories of multi-target and sensors in polar coordinates.

**Figure 5:**
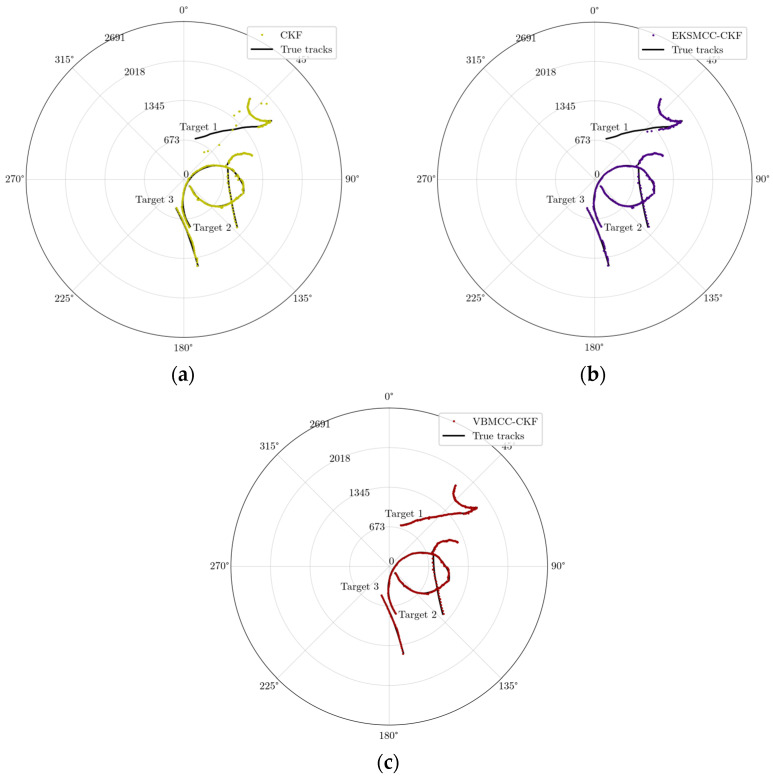
The tracking trajectories estimated by three methods in polar coordinates: (**a**) CKF estimated trajectories; (**b**) EKSMCC-CKF estimated trajectories; (**c**) VBMCC-CKF estimated trajectories.

The motion trajectories of multiple targets and sensor configurations are depicted in Figure 4, while Figure 5 presents representative simulation results for multi-target trajectory estimation under dynamic scenarios. In terms of estimation accuracy, CKF exhibits significant cumulative errors for Target 1, 2, and 3 due to its assumption of a fixed turn rate, which neglects target maneuverability.

EKSMCC-CKF reduces modeling errors by incorporating a time-varying turn rate auxiliary variable and improves tracking performance on the basis of empirical MCC, yet it suffers from target loss under anomalous noise interference. In contrast, by adaptively adjusting the kernel size via variational Bayesian methods in response to disturbance intensity, VBMCC-CKF achieves complete tracking of all targets.

**Figure 6:**
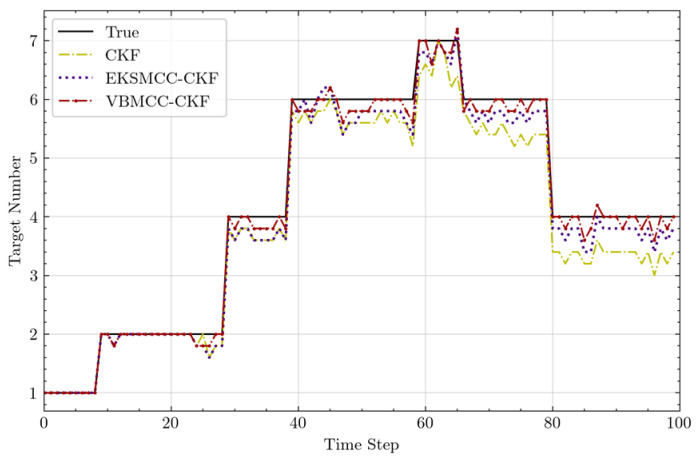
Target number estimated by three methods.

Statistical results for target cardinality estimation robustness are shown in Figure 6. VBMCC-CKF demonstrates superior performance in false alarm control: during anomalous noise intervals, VBMCC-CKF achieves the lowest false alarm rate, whereas CKF exhibits the highest rate due to its susceptibility to accumulated estimation errors, which frequently induce erroneous judgments. Notably, CKF’s false alarm rate even exceeds that of EKSMCC-CKF. In terms of missed detection mitigation, VBMCC-CKF demonstrates a significantly lower mean error in target cardinality estimation compared to the other two methods. This superiority stems from its integration of higher-order innovation terms and an adaptive kernel size adjustment mechanism that dynamically suppresses outliers. Consequently, VBMCC-CKF rapidly converges to stable estimates when targets abruptly appear or disappear, resulting in the lowest missed detection rate. In contrast, CKF, which is highly sensitive to abrupt state transitions, accumulates substantial errors under rapid target population changes, leading to the highest missed detection rate.

**Figure 7:**
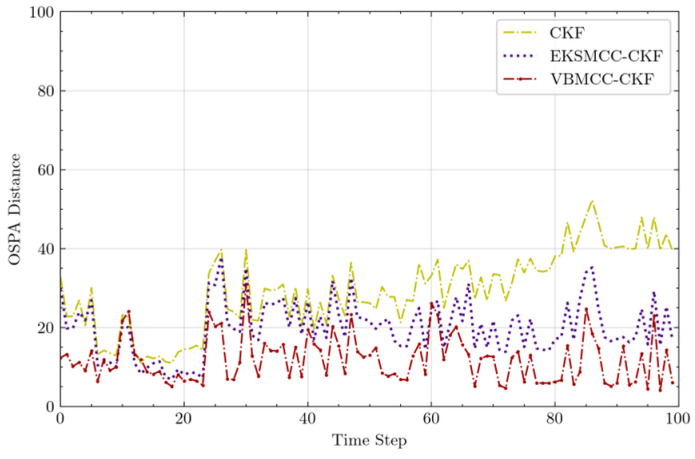
Performance metric for multi-target tracking: OSPA distance.

The comprehensive performance is evaluated via the OSPA distance and the supplementary metrics of HRs and RMSE-TP, as illustrated in Figure 7 and Table 2. VBMCC-CKF demonstrates the smallest fluctuation in OSPA distance, consistently maintaining values below 33 m. Furthermore, it achieves an average OSPA (Avg-OSPA) of 11.897 m, representing a significant reduction of 40% compared to EKSMCC-CKF (19.8291 m) and 59.43% relative to CKF (29.3081 m). During the final maneuver phase, the OSPA distance for CKF surges due to a model mismatch, while VBMCC-CKF, leveraging its time-varying turn rate model and integrating higher-order innovation terms with adaptive kernel scaling, maintains higher estimation accuracy for both target states and cardinality, thus ensuring stable errors. The statistical data for HR and RMSE-TP in Table 2 demonstrate that VBMCC-CKF achieves an RMSE-TP of 11.897 m with 97.39% valid tracking frames and a minimal loss rate of 2.61%. Notably, VBMCC-CKF outperforms the other two methods by attaining the highest HR value, indicating its superior capability to maintain robust tracking under non-Gaussian noise disturbances. Furthermore, its RMSE-TP is the lowest among all compared methods, confirming significantly enhanced estimation accuracy during sustained target tracking. These results collectively validate VBMCC-CKF’s exceptional precision and adaptability in dynamic multi-target scenarios.

The performance discrepancies among the three methods originate from distinct core design philosophies. First, explicit modeling of time-varying turn rates via auxiliary variables ensures motion model completeness, thereby reducing turn rate estimation errors. Second, regarding abnormal disturbance suppression mechanisms, MCC suppresses the influence of measurement outliers through online adaptive kernel size adjustment. Under noise disturbances with a 10-fold covariance (abrupt changes), the state estimation variance is drastically reduced. Finally, in balancing computational efficiency, as shown in Table 2, VBMCC-CKF incurs a marginally higher per-step computational time (0.324 s) compared to EKSMCC-CKF (0.3064 s) due to the added variational Bayesian iterations for kernel size optimization. This demonstrates that robustness enhancements do not impose significant computational overhead.

## 6. Conclusions

In this paper, a novel VBMCC-CKF framework is proposed by integrating higher-order moment information from MCC with a variational Bayesian online alternating optimization framework. By adaptively adjusting the kernel size in response to disturbance intensity, the proposed method addresses the critical challenge of robust state estimation in dynamic systems under non-Gaussian noise environments. On the one hand, VBMCC-CKF enhances robustness against heavy-tailed noise and outliers by implicitly extracting higher-order statistical moments of innovation vectors through the Taylor expansion properties of the MCC Gaussian kernel, surpassing the limitations of traditional MMSE-based approaches. On the other hand, VBMCC-CKF models the kernel size as an inverse-gamma distributed random variable and leverages its conjugate relationship with Gaussian states to formulate a state-kernel size alternating optimization framework, which features a concise structure and rapid convergence within 2–3 iterations. This mechanism realizes the dynamic adjustment of the kernel size by minimizing the KL divergence and approximating the joint posterior distribution, which not only resolves the mismatch issue of the traditional MCC filter’s kernel size selection strategy under abrupt disturbance intensity changes but also incurs only a marginal increase in per-step computational overhead. Finally, the modular architecture of VBMCC-CKF enables seamless integration into multi-Bernoulli filtering frameworks, resolving the multi-target estimation problem in the unified probability space. Extensive validation across single-target and multi-target tracking scenarios highlights its generalized applicability. Thus, a theoretically rigorous adaptive kernel size optimization paradigm is established for non-Gaussian estimation by deeply integrating the variational Bayesian framework with MCC-CKF. Future work will explore autonomous kernel parameter optimization via deep learning to enhance adaptability in dynamic environments and will continue to expand investigations into conjugate distribution generalization, particularly exploring the modeling potential of Gamma and Beta distributions for kernel size adaptation and will then extend the novel filtering architectures based on variational inference to critical application domains, such as GNSS/INS integration.

## Figures and Tables

**Table 1:** The performance evaluation of single-target tracking methods: a comprehensive statistical assessment via complementary metrics.

Method	CKF	MD-ARCKF	FKSMCC-CKF	EKSMCC-CKF	VBMCC-CKF
Average CPU time (s)	0.0059	0.008	0.0078	0.0081	0.0092
Average iterations	—	—	1.96	1.99	2.13
Avg-RMSE (m)	70.4105	40.3779	48.9084	50.6090	34.5919
peak RMSE (m)	137.0961	64.0154	90.6313	68.4827	45.6811

**Table 2:** The performance evaluation of multi-target tracking methods: a comprehensive statistical assessment via complementary metrics.

Method	CKF	EKSMCC-CKF	VBMCC-CKF
CPU time (s)	0.2474	0.3064	0.3240
HR (%)	79.86%	80.33%	97.39%
RMSE-TP (m)	11.2263	9.3477	9.0685
Avg-OSPA (m)	29.3081	19.8291	11.8970

## Data Availability

The original contributions presented in this study are included in the article; further inquiries can be directed at the corresponding author.
